# The Influence of a Metal Stent on the Distribution of Thermal Energy during Irreversible Electroporation

**DOI:** 10.1371/journal.pone.0148457

**Published:** 2016-02-04

**Authors:** Hester J. Scheffer, Jantien A. Vogel, Willemien van den Bos, Robert E. Neal, Krijn P. van Lienden, Marc G. H. Besselink, Martin J. C. van Gemert, Cees W. M. van der Geld, Martijn R. Meijerink, John H. Klaessens, Rudolf M. Verdaasdonk

**Affiliations:** 1 Department of Radiology and Nuclear Medicine, VU University Medical Center, Amsterdam, The Netherlands; 2 Department of Surgery, Academic Medical Center, Amsterdam, The Netherlands; 3 Department of Urology, Academic Medical Center, Amsterdam, The Netherlands; 4 Department of Radiology, The Alfred Hospital, Melbourne, Australia; 5 Department of Radiology, Academic Medical Center, Amsterdam, The Netherlands; 6 Department of Biomedical Engineering and Physics, Academic Medical Center, Amsterdam, The Netherlands; 7 Department of Mechanical Engineering, Eindhoven University of Technology, Eindhoven, The Netherlands; 8 Department of Physics and Medical Technology, VU University Medical Center, Amsterdam, The Netherlands; National Research Council, ITALY

## Abstract

**Purpose:**

Irreversible electroporation (IRE) uses short duration, high-voltage electrical pulses to induce cell death via nanoscale defects resulting from altered transmembrane potential. The technique is gaining interest for ablations in unresectable pancreatic and hepatobiliary cancer. Metal stents are often used for palliative biliary drainage in these patients, but are currently seen as an absolute contraindication for IRE due to the perceived risk of direct heating of the metal and its surroundings. This study investigates the thermal and tissue viability changes due to a metal stent during IRE.

**Methods:**

IRE was performed in a homogeneous tissue model (polyacrylamide gel), without and with a metal stent placed perpendicular and parallel to the electrodes, delivering 90 and 270 pulses (15–35 A, 90 μsec, 1.5 cm active tip exposure, 1.5 cm interelectrode distance, 1000–1500 V/cm, 90 pulses/min), and *in-vivo* in a porcine liver (4 ablations). Temperature changes were measured with an infrared thermal camera and with fiber-optic probes. Tissue viability after *in-vivo* IRE was investigated macroscopically using 5-triphenyltetrazolium chloride (TTC) vitality staining.

**Results:**

In the gel, direct stent-heating was not observed. Contrarily, the presence of a stent between the electrodes caused a higher increase in median temperature near the electrodes (23.2 vs 13.3°C [90 pulses]; p = 0.021, and 33.1 vs 24.8°C [270 pulses]; p = 0.242). *In-vivo*, no temperature difference was observed for ablations with and without a stent. Tissue examination showed white coagulation 1mm around the electrodes only. A rim of vital tissue remained around the stent, whereas ablation without stent resulted in complete tissue avitality.

**Conclusion:**

IRE in the vicinity of a metal stent does not cause notable direct heating of the metal, but results in higher temperatures around the electrodes and remnant viable tissue. Future studies should determine for which clinical indications IRE in the presence of metal stents is safe and effective.

## Introduction

Irreversible electroporation (IRE) is a relatively novel ablation modality that uses electrical energy to induce cell death [1]. Electrodes are placed around a tumor, through which high-voltage, but sub-millisecond electrical pulses are applied at a low frequency (0.5–2 Hz). As opposed to thermal ablation techniques, the electrical pulses are designed to distort the pre-existing cellular membrane potential, leading to disruption of the lipid bilayer, after which the cell loses its homeostatic properties and dies [2–5]. Preclinical studies have shown that within the ablation zone IRE mostly affects cells, leaving the supporting extracellular matrix structures relatively intact [6–8]. This preservation of gross anatomic architecture allows tumors near vascular and biliary structures that are otherwise unresectable or unamenable to thermal-based modalities, to be ablated safely [9,10].

Although IRE was initially introduced as being non-thermal, several studies have now demonstrated that clinical therapies employing high pulse numbers over the electrode pairs (70–200 per pair) in an electric conductive medium inevitably produces cumulative secondary heat due to Joule heating that may affect treatments [11–15], especially in the immediate vicinity of the electrodes where the current density is highest [12,16]. Because IRE is typically used around structures vulnerable to thermal injury, the search for optimal ablation settings minimizing the probability for thermal damage whilst still achieving complete tumor cell death, continues [16,17].

Early clinical application of IRE in hepatopancreatobiliary tumors has raised the question whether thermal injury occurs in the presence of a metal stent, since these patients frequently present with a bare metal Wallstent in situ to resolve obstructive jaundice caused by tumor compression on the common bile duct. These metal stents have a smaller risk of migration, occlusion, therapeutic failure, and cholangitis compared to plastic biliary endoprostheses [18], but can only be removed with extensive surgical or endoscopic manipulation. Given the high electrical and thermal conductivity of metal relative to mammalian tissue, the safety and efficacy to perform IRE in the vicinity of a metal stent has been subject to debate [14,19,20]. The manufacturer of the Nanoknife^®^ electroporation device has stated that the presence of a metal stent within the ablation zone is an absolute contraindication. As a result, many patients with pancreatic or extrahepatic cholangiocarcinoma with a metal stent are withheld IRE treatment.

Recently, a fatal case was published in which several complications following IRE ablation in the pancreatic head region with a metal stent in situ were described, including perforation of the duodenum and transverse colon in close proximity to the stent, and bleeding from a branch of the superior mesenteric artery [19]. In a reply to the published case, we used a mathematical model to calculate the potential effect of a metal stent on heat development, which seemed negligible [21]. Oppositely, a second case was recently published in which IRE was performed successfully around a metal stent for perihilar cholangiocarcinoma [22].

Still, the range and extent of effects of a metal stent within the ablation zone is unknown and requires additional controlled experimental evaluation. Given the great impact of an absolute contraindication, a precise evaluation of the effect of IRE around metal objects is warranted. The purpose of this study was to determine the distribution of thermal energy and the potential clinical implication of IRE around a metal stent using experimental models.

## Materials and Methods

### *In-Vitro* Experiment

**Fig 1:**
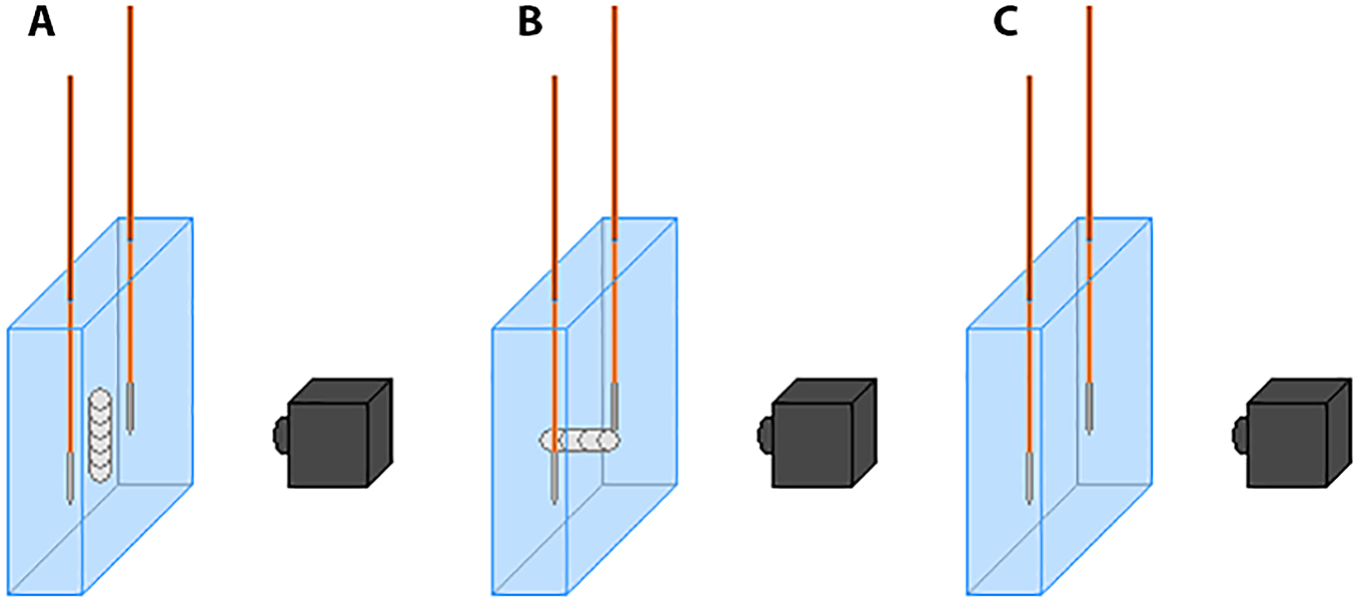
Setup of IRE ablations performed in a tissue phantom. (A) electrodes parallel to stent; (B) electrodes perpendicular to stent; (C) electrodes without stent.

Self-expandable nickel and titanium (nitinol) stents (Epic, Boston Scientific, Marlborough, Massachusetts, US) with a 5 mm diameter, 60 mm length and 0.19 mm mesh thickness were placed inside a transparent gel made of 150 ml saline (NaCl 0.9%), 125 mg ammonium persulfate, 100 ml 30% acrylamide/bis solution and 200 μl tetramethylethyleendiamine, mimicking human soft tissue with respect to electrical and thermal conduction properties [23]. One electrode was placed on each side of and parallel to the metal stent (“stent-IRE”), with an inter-electrode distance (IED) of 1.5 cm, active tip length of 1.5 cm, and 0.5 cm distance to the tissue surface. The same setup was used without a stent between the electrodes (“no-stent-IRE”) (Fig 1). For ablation, the NanoKnife^®^ IRE console (AngioDynamics, Latham, New York, US) was set at 1x90 and 3x90 (270) pulses, with a pulse length of 90 μsec, 90 pulses/minute and a pulse intensity of 1500 V (1000 V/cm voltage-to-distance ratio), aiming at a delivered current of 15–35 Amperes (A). Each experiment was repeated five times. The temperature of the tissue surface was visualized using a Xenics Gobi-384 thermal camera, which records thermal changes of 0.05°C. Temperature data were extracted using the Xeneth software package (Xenics, Leuven, Belgium) [23,24].

### *In-Vivo* Experiment

Following an approved Institutional Animal Ethics Committee protocol, four IRE ablations were performed in the liver periphery of a domestic farm pig (specific pathogen free animals, produced by farm Van Beek B.V., the Netherlands, housed under standard laboratory conditions, weighing approximately 50 kg), with and without stent, with two ablations each for 90 and 270 pulses. The animal was sedated with intramuscular ketamine (10–15 mg/kg), midazolam (1–1.5 mg/kg), and atropine (1.5 ml/50 kg). After intubation, anesthesia was maintained through inhaled isoflurane (2%-4%) and intravenous ketamine (2 mg/kg/h), sufentanil (5–10 mg/kg/h), midazolam (1–2 mg/kg/h), and rocuronium (2–2.5 mg/kg/h). Before IRE, intravenous bolus injections of rocuronium (1–1.5 mg/kg) were administered for complete muscle relaxation. The animal was placed in the supine position and the liver was exposed through a medial laparotomy.

**Fig 2:**
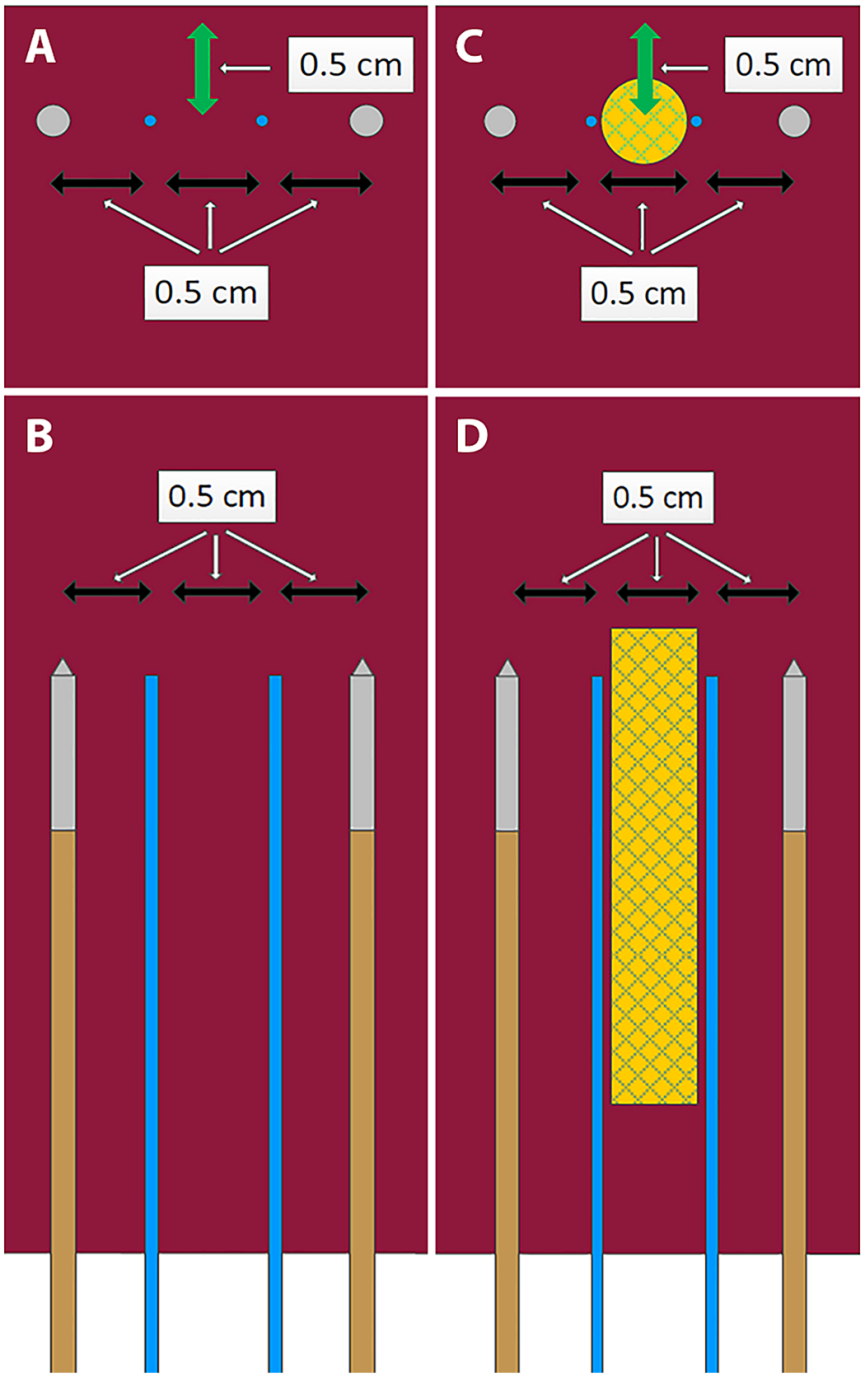
Setup of IRE ablations performed in *in-vivo* porcine liver showing the electrodes (brown/gray) and temperature probes (blue). No-stent-IRE (A, cross-sectional; B, longitudinal) and stent-IRE (C, cross-sectional; D, longitudinal). Green arrow represents the distance to the liver surface.

Stents were placed through a puncture hole and expanded to 0.5 cm diameter parallel to and 0.5 cm beneath the liver surface using ultrasound guidance. Electrodes were positioned parallel to the stent at a distance of 0.5 cm on either side of the stent, corresponding to an IED of 1.5 cm. To measure the temperature within the ablated area, two fiber-optic temperature probes, with a 1 mm diameter (TRUE Lumiterm X5, Ipitek, Carslbad, CA, US) were placed directly against either side of the stent at an equal depth to the IRE electrode tips, registering 0.05°C temperature differences (Fig 2). The same experimental setup for no-stent-IRE was used. The liver surface temperature was measured using the thermal camera mounted to the operating table. Ablations were performed using the same settings as with the *in-vitro* experiments except for the voltage, which was 2250 V (1500 V/cm voltage-to-distance ratio). During electroporation the animal was kept in apnea. Thirty minutes after the last ablation the animal was euthanized by exsanguination. Tissue evaluation consisted of macroscopic examination. Each specimen was sliced into two parts, either parallel or perpendicular to the electrodes. Of each specimen, one half was immediately fixated in formalin. The other half was incubated with 5-triphenyltetrazolium chloride (TTC) vitality staining for 30 minutes at 37°C prior to formalin fixation to identify areas of irreversible cell damage [25,26].

### Analysis and Statistics

The delivered energy per pulse in Joule (J) was calculated using the following formula:

Energy (J)=Voltage (V)xCurrent at first pulse (A)xpulse duration (s)

Continuous variables were presented as median and range. Non-parametric tests were used for non-normally distributed data, where a p-value of <0.05 was considered statistically significant. Data were analyzed using SPSS version 20.0 (IBM statistics, Inc., Chicago).

## Results

### *In-Vitro* Experiment

**Fig 3:**
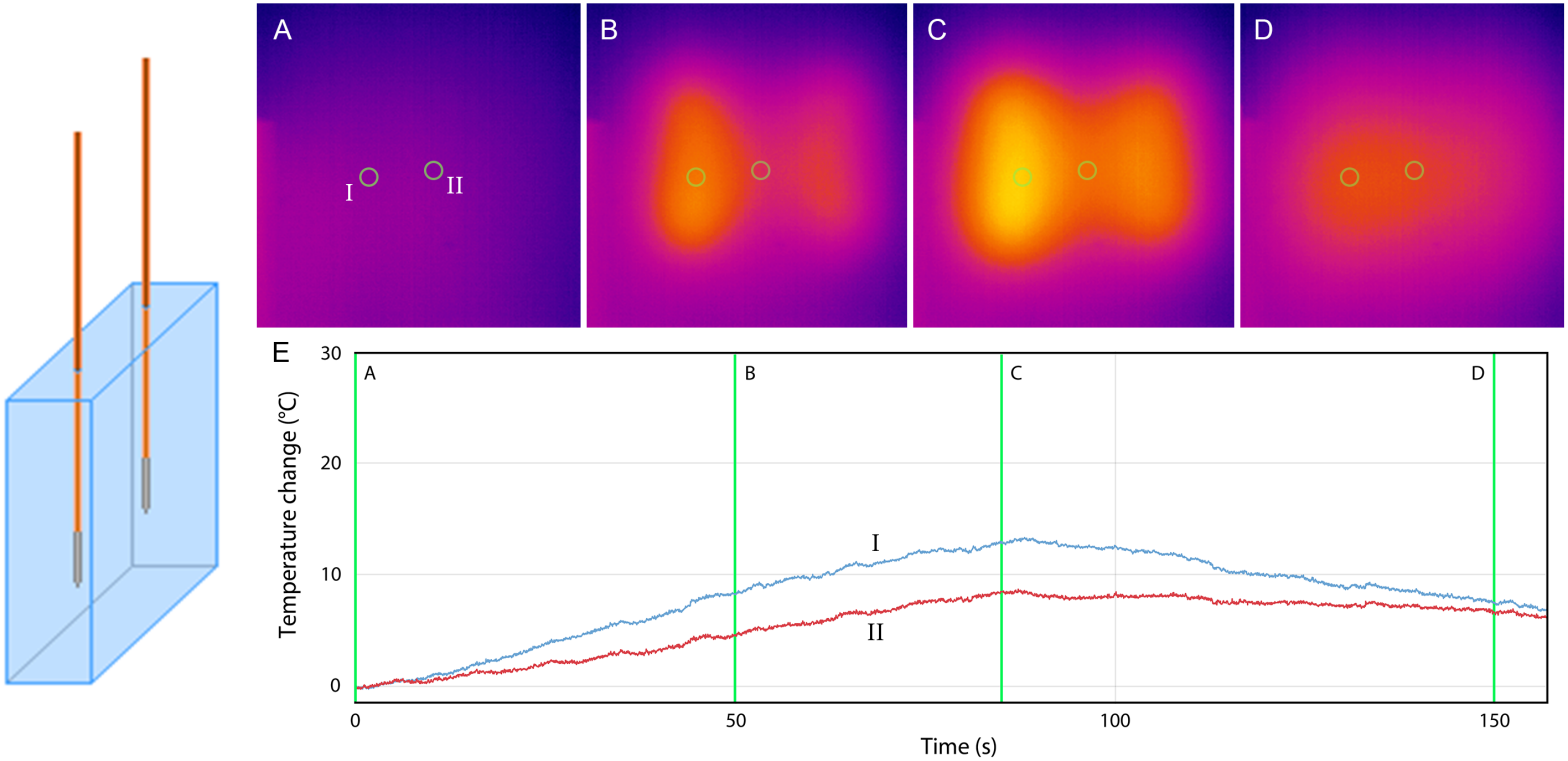
No-stent-IRE. Thermal camera images during 90 pulses (A-D). (E) Graph showing the temperature increase at the surface of the gel 5 mm from (I) the active tip of the electrode and (II) in between the electrodes. (A) pre-IRE (B) after 50 IRE pulses (C) after 90 IRE pulses (D) 75 seconds after last IRE pulse.

**Fig 4:**
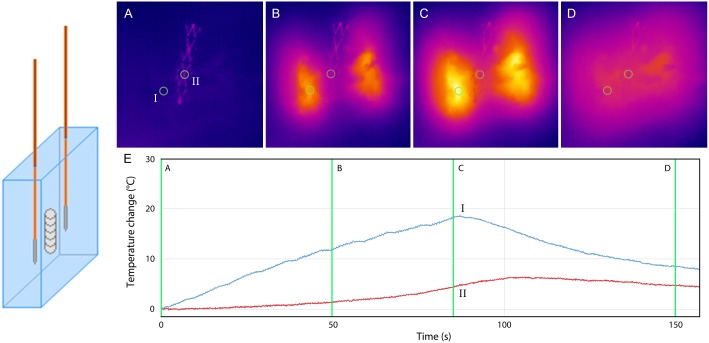
Stent-IRE, parallel. Thermal camera images during 90 pulses (A-D). (E) Graph showing the temperature increase at the surface of the gel 5 mm from (I) the active tip of the electrode and (II) the stent. (A) pre IRE (B) after 50 IRE pulses (C) after 90 IRE pulses (D) 75 seconds after last IRE pulse.

**Fig 5:**
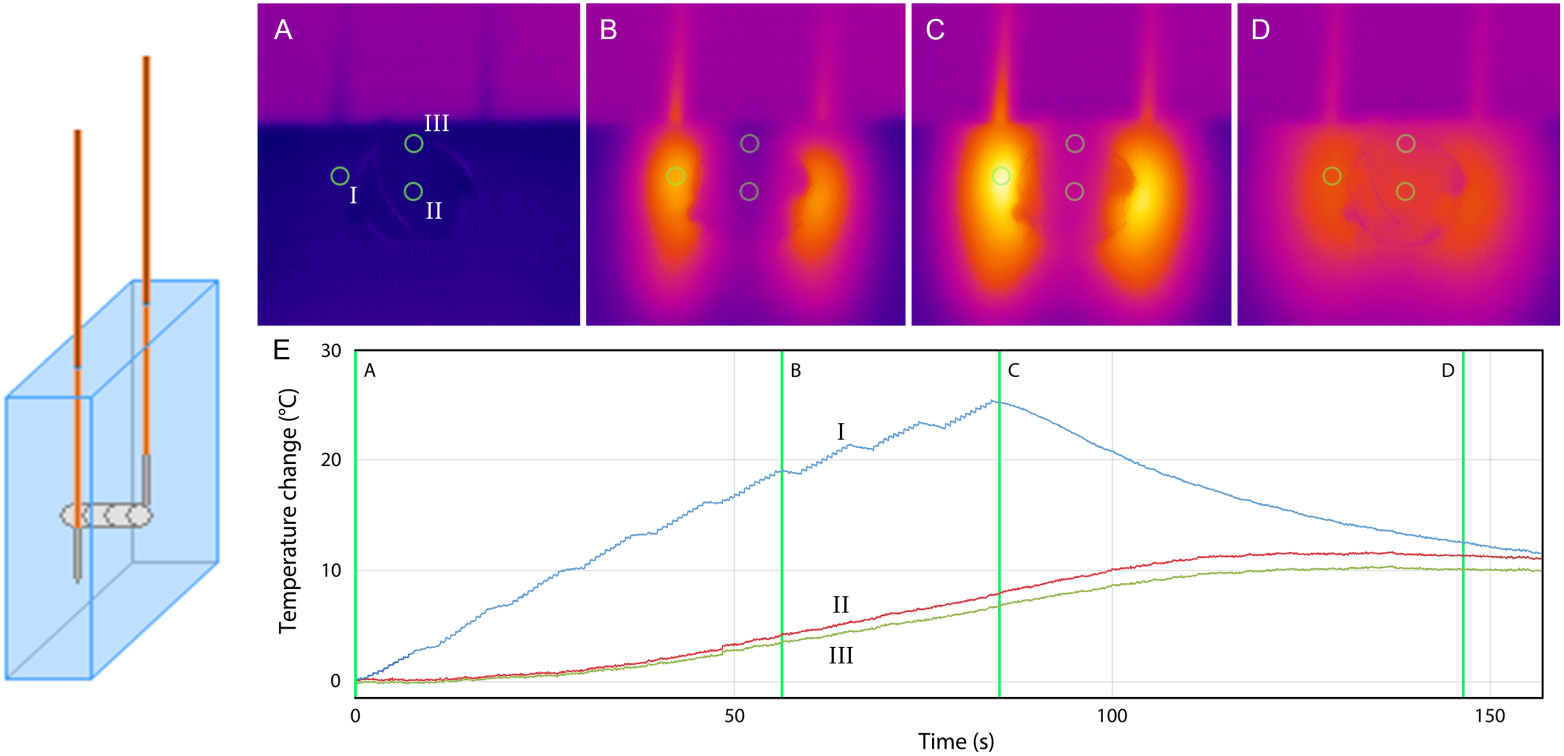
Stent-IRE, perpendicular. Thermal camera images during 90 pulses (A-D). (E) Graph showing the temperature increase at the surface of the gel, 5 mm from (I) the active tip of the electrode, (II) inside the stent and (III) at the margin of the stent. (A) pre-IRE (B) after 60 IRE pulses (C) after 90 IRE pulses (D) 60 sec after the last IRE pulse.

During all *in-vitro* ablations (stent-IRE and no-stent-IRE), a constant temperature rise was detected, which peaked at the electrode tips and gradually decayed to the area between and then away from the electrodes. The highest increase in temperature was always measured at the tip of the electrodes. Temperature increase was larger after 270 pulses than after 90 pulses. Median temperature increases for the different ablation protocols are shown in Table 1; median currents reached are shown in Table 2. Fig 3 shows the representative results of the thermal camera during 90 pulses no-stent-IRE. For stent-IRE, no direct stent-heating was observed (Figs 4 and 5). The maximum temperature increase at the location of the stent in stent-IRE was similar to the same region in no-stent-IRE (p = 0.592 [90 pulses] and p = 0.567 [270 pulses]), but was reached approximately 10–20 seconds later. The maximum temperature increase measured at the tip of the electrodes in stent-IRE was higher than in no-stent-IRE (p = 0.021 for 90 pulses and p = 0.242 for 270 pulses, Figs 4 and 5). Median current at the first pulse of IRE was higher in stent-IRE (p = 0.044, Table 2) as well as current rise, but this difference was not significant (p = 0.266 [90 pulses] and p = 1.000 [270 pulses], Table 3).

**Table 1:** Absolute maximum temperature increase measured between the electrodes and at the tip of the electrodes.

Median maximum increase in temperature in°C (range) in tissue phantom			
	No stent	Stent parallel	Stent perpendicular
Between electrodes			
90 pulses	10.2 (7.4–12.6)	8.6 (6.9–11.5)*	10.7 (9.6–11.7)*
270 pulses	23.6 (17.0–26.7)	21.0 (19.7–23.5)**	22.0 (21.1–24.0)**
Electrode tip			
90 pulses	13.3 (11.6–14.1)	19.4 (14.7–21.3)*	23.2 (19.8–25.2)*
270 pulses	24.8 (17.2–26.6)	26.5 (25.5–35.4)**	33.1 (33.1–38.1)**

Data represent the median and range of 5 experiments.

* 4 experiments, 1 aborted due to high current,

** 3 experiments, 2 aborted due to high current.

**Table 2:** Median current at the first pulse of each ablation in tissue phantom (range).

Current at first pulse in Amperes		Energy dissipation in Joule/pulse
No-stent-IRE	17 (14–18)	2.30 (1.89–2.43)
Stent-IRE	20 (16–22)	2.70 (2.16–2.97)
Mann-Whitney U	0.044	

**Table 3:** Current increase during *in vitro* IRE.

Median current increase in Amperes (range)			
	No-stent-IRE	Stent-IRE	Mann-Whitney U
90 pulses	5 (4–7)	6.5 (5–11)	0.266
270 pulses	10 (8–13)	13 (11–18)	1.000

### *In-Vivo* Experiments

**Fig 6:**
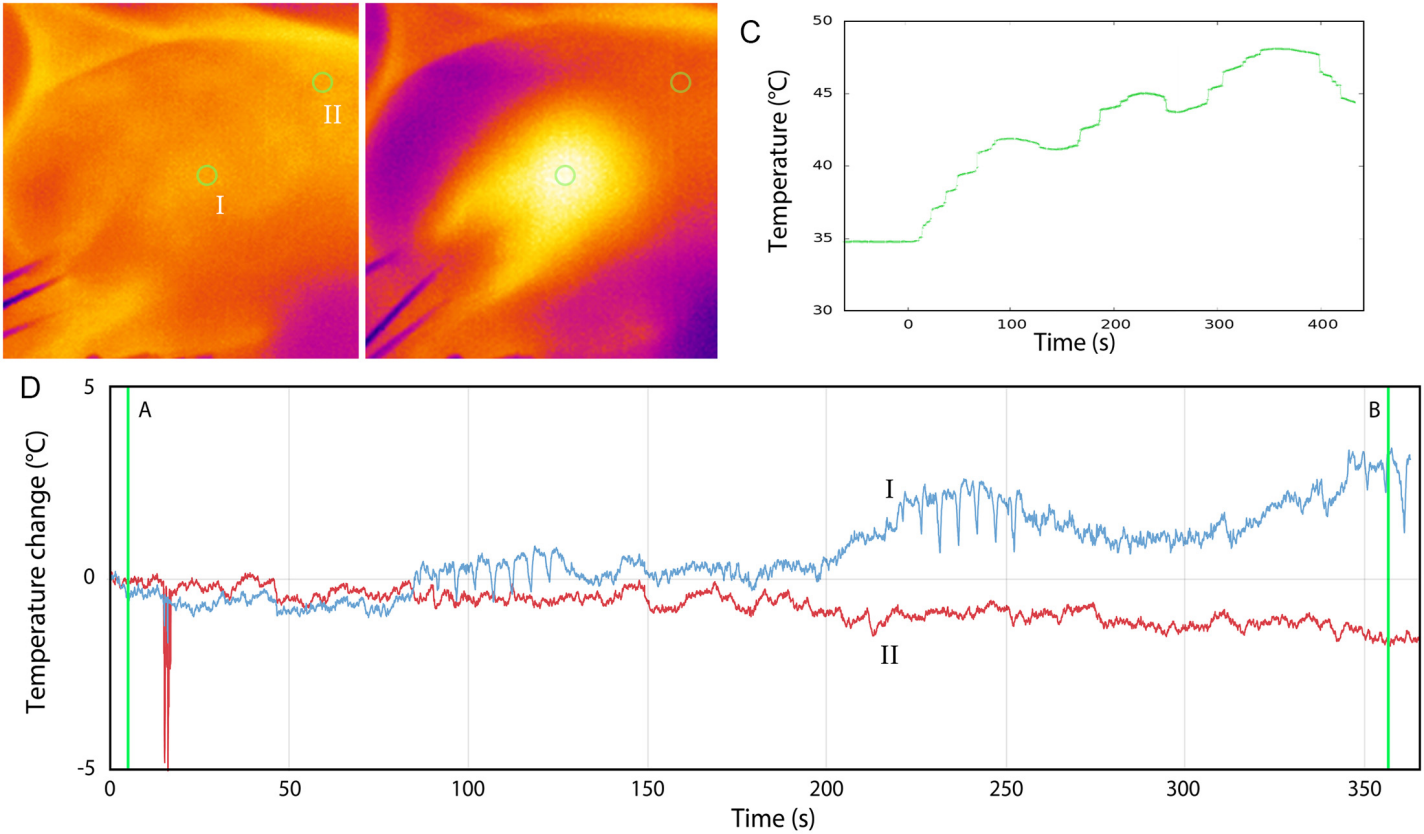
No-stent-IRE. Thermal effects during 270 pulses in porcine liver; Thermal camera showing the temperature increase at the surface of the liver (5 mm from the active tip of the electrode) (A) before and (B) directly after 270 pulses (I: surface above ablation zone, II: surface of normal liver). (C) Increase in surface temperature measured with fiber-optic probes during ablation. (D) Increase in temperature measured with thermal camera during ablation (the disturbance at 100 sec is caused by an ultrasound measurement).

**Fig 7:**
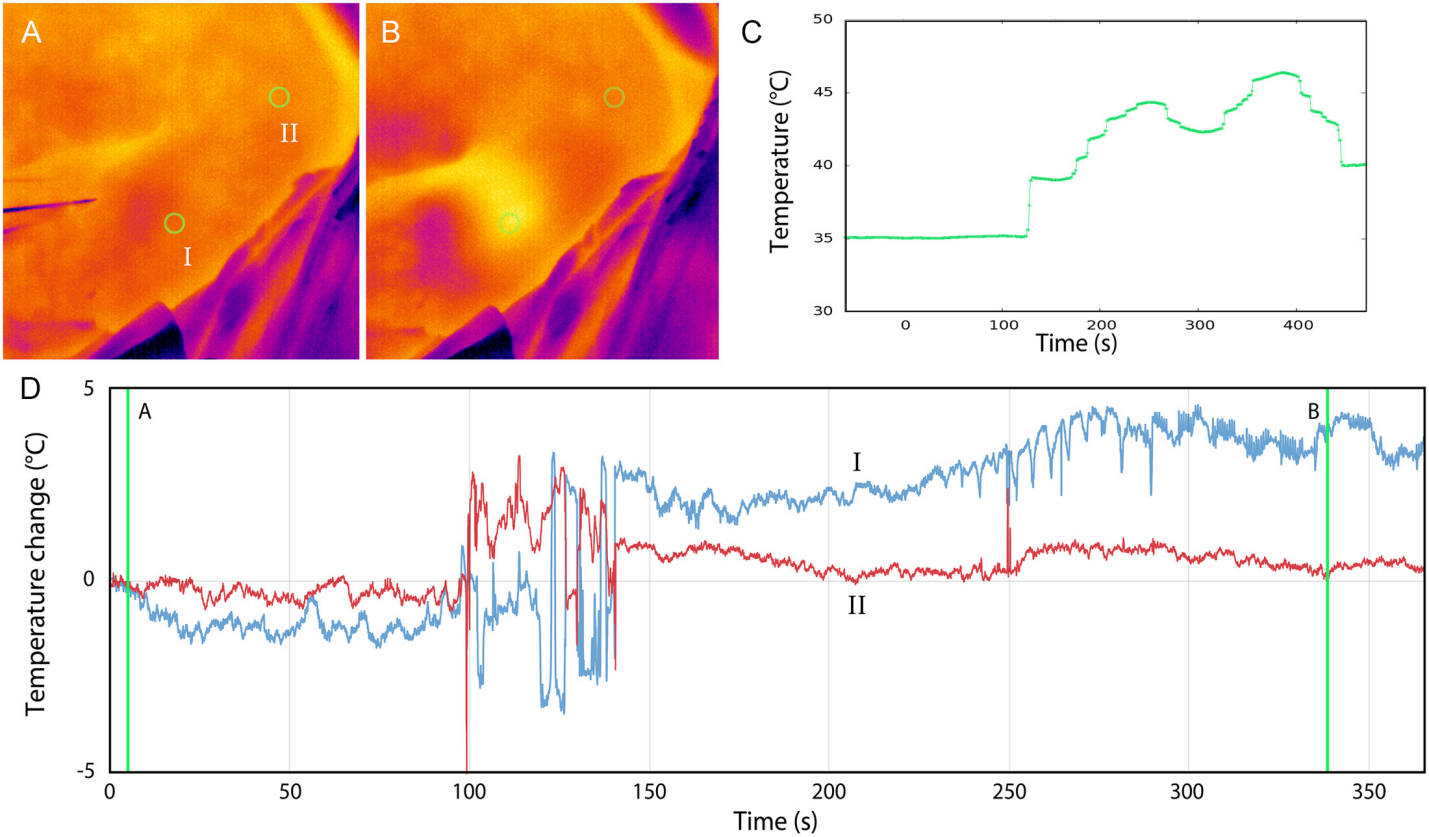
Stent-IRE. Thermal effects during 270 pulses in porcine liver; Thermal camera showing the temperature increase at the surface of the liver (5mm from the active tip of the electrode) (A) before and (B) directly after 270 pulses (I, surface above ablation zone; II, surface of normal liver). (C) Increase in surface temperature measured with thermal camera during ablation. (D) Increase in temperature measured with fiber optic probes during ablation.

**Fig 8:**
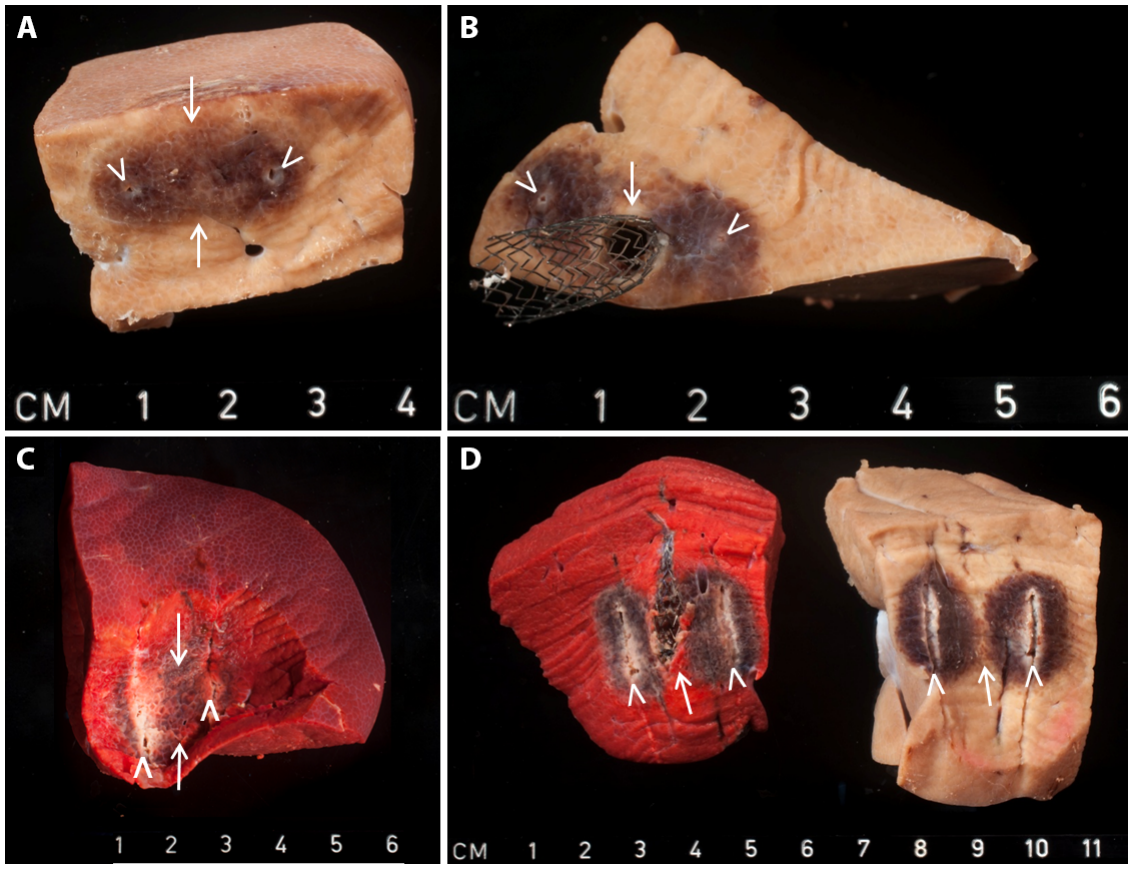
Gross pathology without staining (A, B, D right specimen) and with TTC vitality staining (C, D left specimen) of IRE ablated liver; (A, B) 90 pulses, sliced perpendicular to electrode placement, (A) no-stent-IRE and (B) stent-IRE; (C, D) 270 pulses, sliced parallel to electrode placement, (C) no-stent-IRE and (D) stent-IRE. White arrowheads represent the location of needle placement; white arrows represent the center of the ablation zone.

Temperatures increased during all ablations (Table 4, Figs 6 and 7). In no-stent-IRE, gross pathology showed a homogeneous appearing ablation zone, continuous from one electrode to the other (Fig 8A). Accordingly, TTC vitality staining demonstrated complete avitality of the ablated area around and in between the electrodes visible as a TTC-negative, unstained area (Fig 8C), compared to the vital TTC-positive (red stained) liver tissue distant from the ablation zone. Stent-IRE resulted in an inhomogeneous ablation zone (Fig 8B and 8D) with an area of viable liver tissue immediately surrounding the stent. No coagulative necrosis was noted in the area adjacent to the stent. White coagulation, representing coagulative necrosis caused by thermal damage, was only observed in the immediate vicinity of the electrodes, in both stent-IRE and no-stent-IRE.

**Table 4:** Temperature increase measured by the thermal camera and fiber optic probes for ablation in porcine liver.

Maximum increase in temperature in°C *in-vivo*		
	No-stent-IRE	Stent-IRE
Camera		
90 pulses	1.6	2.0
270 pulses	2.5	3.6
Fibers		
90 pulses	5.1	4.1
270 pulses	11.2	13.3

## Discussion

It was never disputed that every electric field, including a field for irreversible electroporation, produces a thermal effect [17]. Yet, from 2005 onwards, scientists showed that IRE could be isolated from thermal effects and used by itself to produce substantial volumes of tissue ablation *in vivo*, with negligible thermal effects [3,8,27–29]. The application of IRE for the therapeutic ablation of tumors however, has evolved to use more aggressive energy regimens, with higher voltage and higher pulse number protocols. These high-energy regimens have shown to generate potentially harmful thermal effects [11,15,16,30], which is reaffirmed in the present study. Much effort should therefore be put in the development of clinical pulse protocols that mitigate these thermal effects and maintain IRE as the vastly predominant modality of tissue death.

Due to the relative infancy of clinical IRE and the presumably heterogeneous energy distribution resulting from metallic stents in the treatment region, major concerns have been raised about the use of IRE in proximity to metal stents. Chiefly, the presumed heating of the stent and its surroundings as a result of its high electrical conductivity are a concern [14,19,20]. This study demonstrated that the temperature of the stent itself does not exceed the temperature of the adjacent tissue during IRE, implying that there is no direct heating of the stent and that the absolute contraindication in this respect is unsubstantiated. This corresponds to our previous calculations [21]. On the other hand, two different effects were detected which warrant further exploration and consideration in regard to their influence on IRE outcomes: 1) a higher temperature increase around the electrodes and 2) a remnant viable rim immediately surrounding the stent.

A higher temperature around the electrodes is important when considering the location of electrode placement. Usually electrodes are placed within and around the tumor, including vital tissue where vulnerable structures such as bile ducts, nerves, and non-tumorous vessels traverse. Due to the increased heat development around the electrodes, the risk of damage to these structures is increased. This stresses the essence of calculative and precise electrode placement.

The remaining rim of vital liver tissue immediately surrounding the stent is disconcerting, since it may negatively influence oncological outcome. This concern especially relates to tumors in direct contact with the stent—such as perihilar cholangiocarcinomas and other liver tumors. IRE may still be of value when treatment is mainly based on palliation, since tumor debulking may prolong stent patency, thereby reducing disease-related morbidity and postpone tumor progression. From a different perspective, the vital rim could also be considered advantageous. In cases where the stent is placed in a non-invaded bile duct, the 1 mm vital rim can be considered additional protection of the damage-susceptible bile duct. For each individual case of IRE around a stent, the possibilities and limitations should therefore be deliberated cautiously.

In the fatal case published by Månsson *et al* [19], the causality between the stent and the complications could not be established. While this serious complication is indisputably alarming, our results show that direct heating of the stent should not have been the cause. However, the increased temperature surrounding the electrodes may have contributed to the development of the complications, especially if one of the electrodes was placed near the duodenal wall or a large vessel. Neal and colleagues showed no difference in electrical behavior between ablations with and without symmetrically arranged expired radiotherapy seeds in a non-animal model as well as in *ex-* and *in-vivo* canine prostate. Similarly, further in-silico evaluations predicted no significant alteration of the electric field and temperature development [20]. As opposed to the presented study, the tissue adjacent to the seeds within the realm of ablation had the same appearance as the ablated prostate tissue without seeds. The authors stated that larger implants like stents might exert a larger effect on current distribution around the electrodes, which would accord with our results. Grounded metal plates near or within the ablation area can result in large regional changes in electric field distribution, by pulling the electric field away from the positive electrode, shown by Ben David *et al* [1]. However, this pull will not occur with electrically isolated metal objects, like stents. Compared to our findings, Dunki-Jacobs *et al* measured a larger maximum temperature difference of 18°C at 0.5 cm distance from the electrodes, between IRE with and without stent and metal clips placed deep in the porcine liver [14]. However, details about number of electrodes and pulses used, and whether the results referred to stents or clips were not provided. Remarkably, an ineffective ablation was reported with a clip, suggesting a significantly changed electric field distribution. Although this could correlate with our findings, the exact details were not provided.

Our study has several limitations. First, our *in vitro* absolute temperature measurements should be interpreted as describing important trends only and are not representative for the temperatures achieved in perfused tissue, because 1. the camera measures the surface temperature rather than the exact stent, tissue or electrode temperatures, 2. living tissue has a higher baseline temperature and will therefore result in a smaller temperature gradient, and 3. living tissue is perfused and will conduct temperatures faster and further away from the ablation zone compared to the gel, which may explain why we observed smaller temperature changes *in vivo*. Another limitation is the interval from ablation to tissue harvesting, which may have been too short to allow completion of IRE cell death processes. Since the specimens were harvested approximately 2–3 hours after the ablation, the effect of cell death may not have been maximally present at the time of evaluation and our macroscopic findings may have been an underestimation of the actual zone of cell death [25]. Furthermore, we did not account for the physical effect of metal stents in (often infected) biliary obstruction or cancerous tissue, which alters the cellular and stromal tissue aspects, and introduces uncertainties regarding the electrical properties [20]. Finally, the animal experiments were only performed four times and should therefore be interpreted with caution.

The assumption that the metal of the stent would be directly heated in the electrical field of the electrodes is improbable since metal is a good thermal and electrical conductor. However, it can be expected that the metal stent will highly distort the distribution of the electrical field and the pathway of the current between the electrodes—performing similarly to a Faraday’s cage—leading the current around rather than through the stent.

The higher temperature increase around the electrodes can be explained by the lower net resistance in the area of the stent. Given a constant voltage, the net current will increase as a result of decreasing resistance. Indeed, our measurements showed a 15% higher current at the first pulse in stent-IRE compared to no-stent-IRE (p = 0.044). Consequently, more energy is deposited (*P* = *V* x *I = I*^*2*^ x *R*), resulting in a higher temperature increase around the electrodes.

In light of the fact that the actual mechanisms of cell death from IRE remain a topic of discussion by some regarding the potential role of thermal coagulation in the ablation process, multiple hypotheses for the rim of viable tissue around the stent can be surmised. One way or the other, the metal stent will distort both the electrical and thermal field distribution, resulting in an unpredictable ablation zone that apparently results in a small rim of vital tissue near the stent. This could either be ascribed to a less effective IRE effect due to the distorted electrical field, or to a heat-sink effect of the stent if thermal effects would have a substantial role in the mechanism of action. Future experiments should provide more insight in the contribution of both mechanisms.

Current literature on the influence of metal objects remains limited and future work should further characterize the effects. We are currently preparing an animal study in which the findings from this work will be verified and further analyzed. In the meantime, we advise that whenever possible, placement of an uncovered Wallstent should be avoided and a retrievable plastic endoprosthesis or covered endoscopically retrievable Wallstent should be placed instead. Also, in open procedures the stent should be removed peroperatively prior to IRE [31]. Still, for patients in which the stent cannot be removed or in which removal imposes a significant risk, IRE may be considered.

## Conclusion

IRE in the vicinity of a metal stent does not cause notable increased heating of the metal stent, but results in higher temperatures around the electrodes. *In vivo*, a remnant viable tissue region immediately adjacent to the stent was observed. These findings reinforce the appeal to either place plastic biliary endoprostheses or to remove of metal stents prior to IRE whenever possible. Future studies should determine for which clinical indications IRE in the presence of metal stents is safe and effective.
